# ZFN-Mediated *In Vivo* Genome Editing Corrects Murine Hurler Syndrome

**DOI:** 10.1016/j.ymthe.2018.10.018

**Published:** 2018-11-01

**Authors:** Li Ou, Russell C. DeKelver, Michelle Rohde, Susan Tom, Robert Radeke, Susan J. St. Martin, Yolanda Santiago, Scott Sproul, Michael J. Przybilla, Brenda L. Koniar, Kelly M. Podetz-Pedersen, Kanut Laoharawee, Renee D. Cooksley, Kathleen E. Meyer, Michael C. Holmes, R. Scott McIvor, Thomas Wechsler, Chester B. Whitley

**Affiliations:** 1 Gene Therapy Center, University of Minnesota, Minneapolis, MN, USA; 2 Sangamo Therapeutics, Inc., 501 Canal Boulevard, Richmond, CA, USA; 3 Center for Genome Engineering, Department of Genetics, Cell Biology and Development, University of Minnesota, Minneapolis, MN, USA; 4 Research Animal Resources, University of Minnesota, Minneapolis, MN, USA

**Keywords:** gene editing, gene therapy, lysosomal diseases

## Abstract

Mucopolysaccharidosis type I (MPS I) is a severe disease due to deficiency of the lysosomal hydrolase α-L-iduronidase (IDUA) and the subsequent accumulation of the glycosaminoglycans (GAG), leading to progressive, systemic disease and a shortened lifespan. Current treatment options consist of hematopoietic stem cell transplantation, which carries significant mortality and morbidity risk, and enzyme replacement therapy, which requires lifelong infusions of replacement enzyme; neither provides adequate therapy, even in combination. A novel *in vivo* genome-editing approach is described in the murine model of Hurler syndrome. A corrective copy of the *IDUA* gene is inserted at the albumin locus in hepatocytes, leading to sustained enzyme expression, secretion from the liver into circulation, and subsequent uptake systemically at levels sufficient for correction of metabolic disease (GAG substrate accumulation) and prevention of neurobehavioral deficits in MPS I mice. This study serves as a proof-of-concept for this platform-based approach that should be broadly applicable to the treatment of a wide array of monogenic diseases.

## Teaser

*In vivo* genome editing following a single injection of ZFN and IDUA donor encoding AAV8 resulted in metabolic correction and neurological benefit in a murine model of MPS I. These results enable a currently open clinical trial to evaluate targeted genome engineering for MPS I in humans.

## Introduction

Mucopolysaccharidosis type I (MPS I) is a rare autosomal recessive disorder, caused by deficiency of the enzyme α-L-iduronidase (IDUA), which is required for the lysosomal degradation of the glycosaminoglycans (GAGs) dermatan and heparan sulfate.^1^ MPS I patients experience respiratory and cardiac disease, organomegaly (hepato- and splenomegaly), blinding corneal opacification, debilitating joint stiffness, and skeletal deformities (including severe spine deformities).^2^, ^3^ In the most severe phenotype, Hurler syndrome, infants show progressive developmental delay and die between 5 and 10 years of age. The current standard of care for infants with Hurler syndrome is hematopoietic stem cell transplantation (HSCT), which carries a significant risk of mortality (10%–15%) and severe morbidity; if such allogeneic transplant is successful, children may survive into adulthood but still suffer persistent and progressive skeletal deformity and require ongoing surgical interventions. Those affected with more attenuated phenotypes (Hurler-Scheie syndrome and Scheie syndrome) may receive palliative enzyme replacement therapy (ERT).^2^, ^4^ However, despite weekly life-long ERT infusions, affected individuals still incur devastating systemic problems requiring multiple surgical interventions (i.e., hip replacement, cornea transplantation) and suffer a poor quality of life. ERT has negligible neurological impact limited by the blood-brain barrier (BBB) and has little therapeutic effect on cardiac valve disease or skeletal abnormalities.^2^

Site-specific *in vivo* genome editing using engineered zinc finger nucleases (ZFNs) delivered via adeno-associated viral (AAV) vectors is a promising technology for the treatment of monogenic disease.^5^, ^6^, ^7^ One view of therapeutic genome editing is to correct the disease-causing mutation at the endogenous locus. However, individual mutations are patient specific and a broad heterogeneity of mutations can exist among patients within a given disease. Among MPS I patients, although the W402X and Q70X mutations may account for greater than 50% of mutations found in the Caucasian population, this incidence varies widely across ethnic backgrounds and it is well established that significant mutational heterogeneity exists.^8^, ^9^, ^10^ Additionally, depending on the promoter strength at the disease locus, a large proportion of alleles may need to be edited in order to drive therapeutic levels of the corrected protein. Finally, mutation-specific correction may require a new set of targeting reagents (nucleases and corrective transgene donor) to be generated for each disease allele, which may be both cost and time prohibitive. As an alternative, more universal approach, we have previously demonstrated that the liver albumin locus functions as an efficient “safe harbor” site for the insertion and expression of genes that are mutated in a variety of monogenic diseases.^7^ Albumin is an ideal “safe harbor” locus in hepatocytes, due to the ease of liver targeting by AAV relative to other tissues, the liver-specific expression of albumin, and the high level of transcriptional activity of the albumin promoter machinery.^11^ From a practical standpoint, this means that only a small number of albumin alleles may need to be modified by insertion of the corrective transgene in order to drive sufficient therapeutic protein expression, due to the relative promoter strength of albumin as compared to the disease locus in question. Additionally, by selecting a single safe harbor site, the same pair of ZFNs can be utilized to insert different corrective transgenes for corresponding diseases, thereby leveraging a single pair of highly optimized nucleases.

Previous studies have demonstrated the expression of therapeutic transgenes from the albumin locus in the liver;^7^ here, we test the therapeutic benefit of inserting a human IDUA (hIDUA) transgene at the albumin locus in hepatocytes using the murine model of Hurler syndrome.^12^ Following expression from the albumin locus, hIDUA will be targeted for secretion by the endogenous albumin signal peptide and be distributed systemically by the circulation for uptake and cross-correction of GAG substrate degradation in secondary tissues. This one-time treatment would represent a significant improvement over current ERT therapy, which requires lifelong infusions of replacement enzyme. Further, insertion of hIDUA at the albumin locus should eliminate the issue of vector washout associated with episomal AAV gene therapy,^13^, ^14^, ^15^ thereby enabling the long-term treatment of pediatric patients. The results presented here demonstrate the effectiveness of this approach and its potential as a therapeutic strategy for treatment of the mucopolysaccharidoses. Clinical testing of this approach was recently initiated (ClinicalTrials.gov, NCT02702115) for the evaluation of *in vivo* genome editing in humans for the treatment of MPSI.

## Results

### Insertion of hIDUA at the Albumin Locus and Expression in Hepatocytes

**Figure 1:**
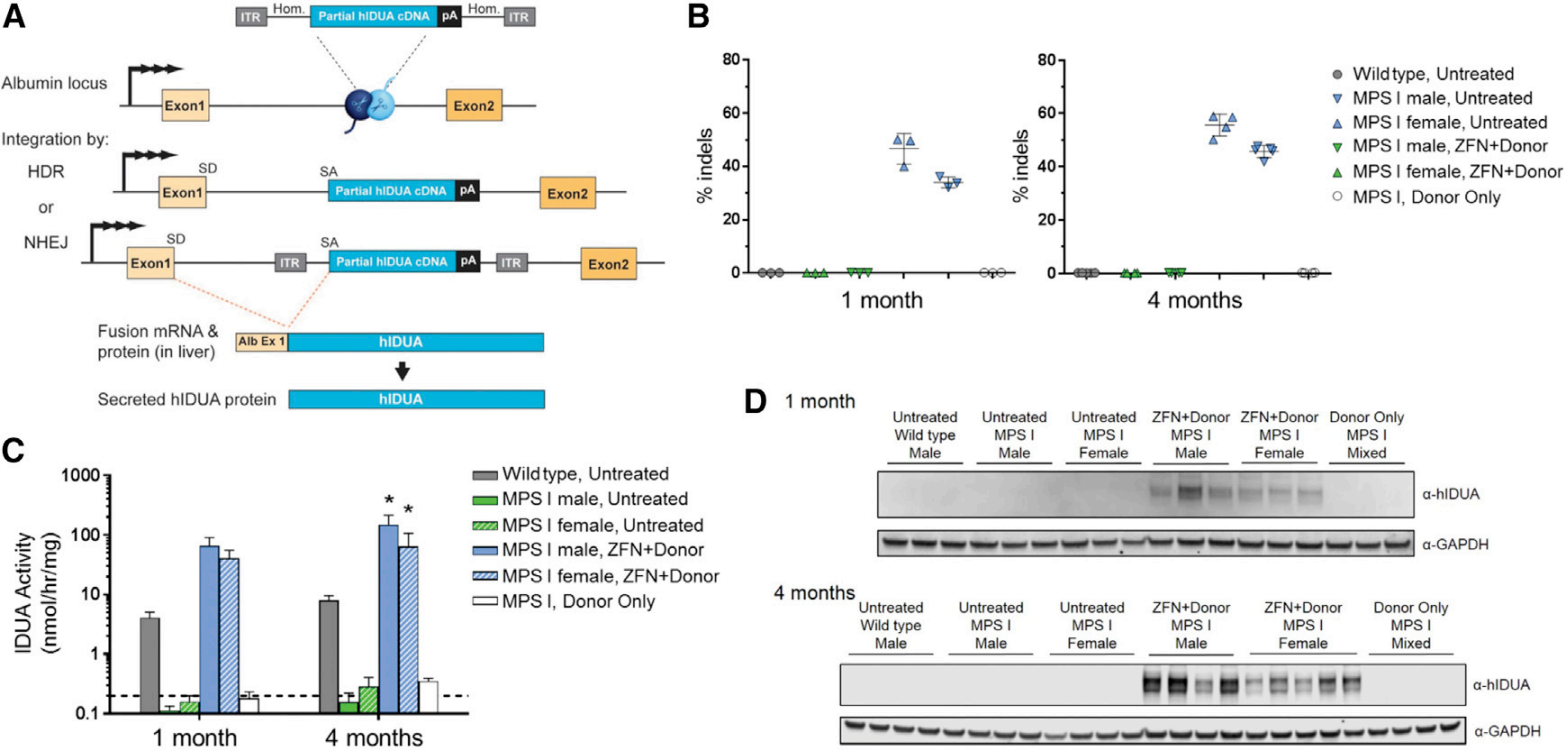
*In Vivo* ZFN-Mediated Insertion of hIDUA at the Albumin Locus Results in Expression of Active Enzyme in MPS I Mouse Hepatocytes (A) Outline of targeting strategy at intron 1 of albumin. Hom, arms of homology to mouse albumin locus; pA, polyadenylation sequence; SD, splice donor site; SA, splice acceptor site. (B) Percent insertion and deletions (% indels) at the ZFN target site in mouse liver 1 or 4 months post-treatment. Each symbol represents an individual mouse, and mean ± SD is shown. (C) IDUA enzyme activity in protein extracts from mouse liver tissue 1 or 4 months post-treatment. Dashed line indicates approximate limit of detection for the assay (0.2 nmol/hr/mg). Data are shown as mean ± SD. *p < 0.05, when compared to the gender-matched untreated MPS I group. n = 3 per group at 1 month and n = 4–5 per group at 4 months. (D) Expression of hIDUA in mouse liver protein extracts 1 or 4 months post-treatment. Each lane represents an individual mouse. GAPDH is shown as a loading control.

The proposed strategy is outlined in Figure 1A, in which a pair of ZFNs driven by a liver-specific promoter-enhancer (the human α-1-antitrypsin [hAAT] promoter and human apolipoprotein [ApoE] enhancer) induce the targeted insertion of a promoterless, partial hIDUA cDNA with the signal peptide removed. A secretory signal peptide is provided by exon 1 from the endogenous albumin locus, which is spliced in-frame with hIDUA following transcription from the endogenous albumin locus. The albumin signal peptide is then cleaved from the final protein product prior to secretion. Although insertion can occur via either the homology-directed repair (HDR) or non-homologous end joining (NHEJ) pathways, the splice acceptor signal present on the donor ensures that the same mRNA and protein species is produced, regardless of the mechanism of insertion.

To test the feasibility of this approach in the murine model of Hurler syndrome, male and female mice were injected via the tail vein with 1.5 × 10^11^ vector genomes (vg) of each AAV2/8 ZFN targeting intron 1 of the albumin locus and 1.2 × 10^12^ vg of an AAV2/8 hIDUA promoterless donor construct and evaluated over 4 months. This *IDUA* knockout model shows the characteristic features of the most severe form of the human disease Hurler syndrome, i.e., craniofacial deformity, learning defects, and increased GAG levels in tissues and urine.^16^, ^17^ Affected mice show visible manifestation at 3–5 weeks, and by 12–16 weeks of age extensive lysosomal accumulation of GAG is evident in the liver and CNS. In addition, affected MPS I mice show cognitive impairments by 5 months of age. This study used 4- to 10-week-old MPS I mice and was based on a disease prevention paradigm, wherein mice in the early stage of disease were administered ZFNs and hIDUA donor and evaluated longitudinally.

The delivered ZFNs were highly active in generating small insertions and deletions (indels) at the albumin target site in mouse liver, leading to 34%–47% indels at 1 month and 46%–56% indels at 4 months post-treatment (Figure 1B). ZFN activity at both 1 and 4 months was slightly higher in male than female treated mice, which may be due to the increased transduction efficiency of the male liver by AAV observed in mice.^18^ ZFN activity led to efficient targeted insertion of the hIDUA transgene at intron 1 of the albumin locus and subsequent production of hIDUA enzyme, as demonstrated by analysis of IDUA enzyme activity in liver tissue from these mice (Figure 1C). Liver IDUA activity in ZFN+donor-treated mice was 10- to 16-fold greater than activity in control wild-type mice at 1 month post-dosing, and a similar fold increase was observed at 4 months, demonstrating the stability of hIDUA insertion and expression from the endogenous albumin locus following a single injection. Expression of hIDUA in the livers of ZFN+Donor-treated mice was further confirmed by western blot using a human-specific IDUA antibody (Figure 1D). hIDUA expression was observed in all ZFN+donor-treated mice at both 1 and 4 months post-dosing, but not in any mice treated with the hIDUA donor construct in the absence of ZFNs (donor only) nor in control wild-type mice, confirming the necessity of both ZFNs and the donor for efficient insertion and expression of the hIDUA transgene.

### Durable Secretion of IDUA from Hepatocytes into Circulation

**Figure 2:**
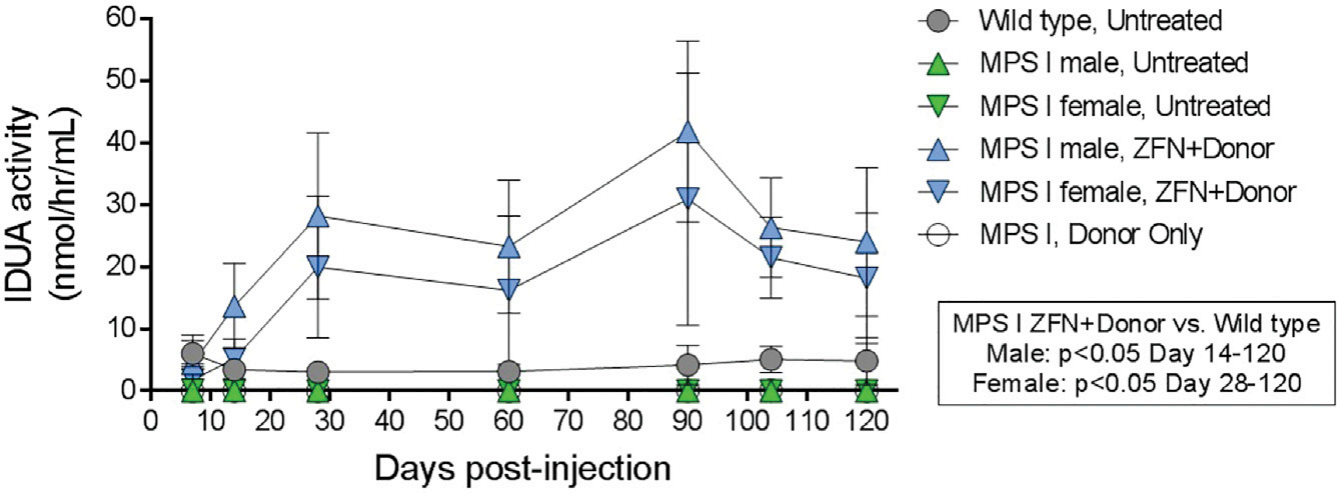
Sustained IDUA Plasma Activity in ZFN+Donor-Treated MPS I Mice IDUA enzyme activity in plasma is shown as mean ± SD. n = 7 ± 8 per group from days 0 to 28 and n = 4 to 5 per group through day 120. IDUA activity in ZFN+Donor treated mice is significantly (p < 0.05) higher than levels found in wild-type mice from day 14 (male mice) or day 28 (female mice) through day 120.

In order to determine whether the hIDUA being produced from the albumin locus of ZFN+donor-treated mice was efficiently secreted into circulation, we assessed plasma IDUA activity at multiple time points over the course of this study. In both male and female ZFN+donor-treated mice, IDUA activity was detectable in the plasma by day 7, surpassed IDUA activity found in wild-type mice by day 14, and continued to increase through day 28 (Figure 2). IDUA plasma activity was 7 (female)- to 9 (male)-fold higher than activity observed in wild-type mice on day 28, which represented a significant increase that was maintained throughout the 120-day study, further demonstrating the durability of this approach. No significant plasma IDUA activity was observed in mice receiving the hIDUA donor in the absence of ZFNs (donor only) or in control, untreated MPS I mice.

### Uptake of IDUA by Secondary Tissues

**Figure 3:**
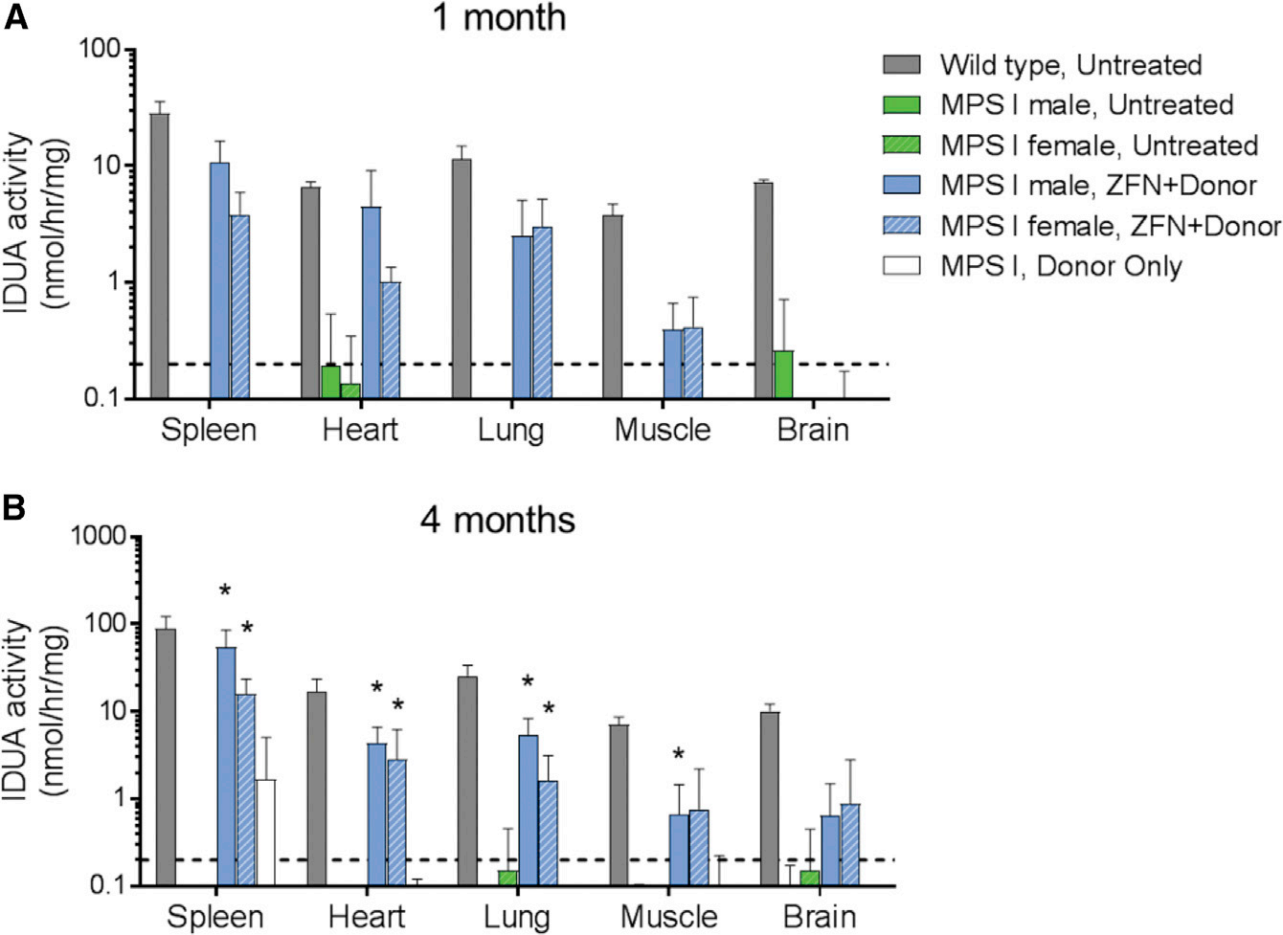
Increased IDUA Enzyme Activity in Secondary Tissues of ZFN+Donor-Treated MPS I Mice IDUA enzyme activity in protein extracts from the listed secondary tissues at 1 (A) or 4 (B) months post-treatment. Data are shown as mean ± SD. Dashed line indicates approximate limit of detection for the assay (0.2 nmol/hr/mg). *p < 0.05, when compared to the gender-matched untreated MPS I group. n = 3 per group at 1 month and n = 4–5 per group at 4 months.

To examine whether the hIDUA being secreted into the bloodstream of ZFN+donor-treated mice was capable of being taken up by secondary tissues, we next examined IDUA enzyme activity in a variety of tissues in these mice. The promoters regulating both ZFN and endogenous albumin expression are not active in these non-hepatic tissues, and so any IDUA activity observed must be due to uptake of hIDUA from circulation following secretion from the liver. The liver-specific activity of the ApoE/hAAT enhancer and promoter that drive ZFN expression was additionally confirmed in a separate study in wild-type mice, in which no ZFN activity was observed in tissues outside of the liver (Figure S1). At 1 month post-treatment in the present study, IDUA activity was clearly increased in the spleen, heart, lung, and muscle (quadriceps) of ZFN+donor-treated mice (Figure 3A). Significantly increased IDUA activity was also observed at 4 months, reaching up to 61% (spleen), 26% (heart), 21% (lung), and 11% (muscle) of wild-type IDUA activity (Figure 3B). A small but non-significant increase in IDUA activity was also observed at 4 months in the brains of both male and female ZFN+donor-treated mice, corresponding to approximately 0.5%–1% of activity found in wild-type mice. These results demonstrate that IDUA produced from the liver albumin locus contains the appropriate modifications, such as mannose-6-phosphorylation, necessary for uptake into secondary tissues. The dependence of hIDUA produced from the albumin locus on the mannose-6-phosphate (M6P) receptor for uptake was further investigated *in vitro*. ZFNs targeting intron 1 of the human albumin locus were developed and used to insert hIDUA in the human hepatoma line HepG2. Subclones actively secreting hIDUA from the albumin locus were derived, and hIDUA-containing supernatant from these cells were incubated with naive secondary target cells. Analysis of IDUA activity in target cells demonstrated that excess, free M6P was able to block hIDUA uptake by secondary cells (Figure S2). Combined, these results demonstrate that hIDUA produced from the albumin locus is efficiently taken up by secondary tissues and uptake is dependent upon the M6P receptor.

### Normalization of GAG Storage in the Liver and Secondary Tissues of Treated Mice

**Figure 4:**
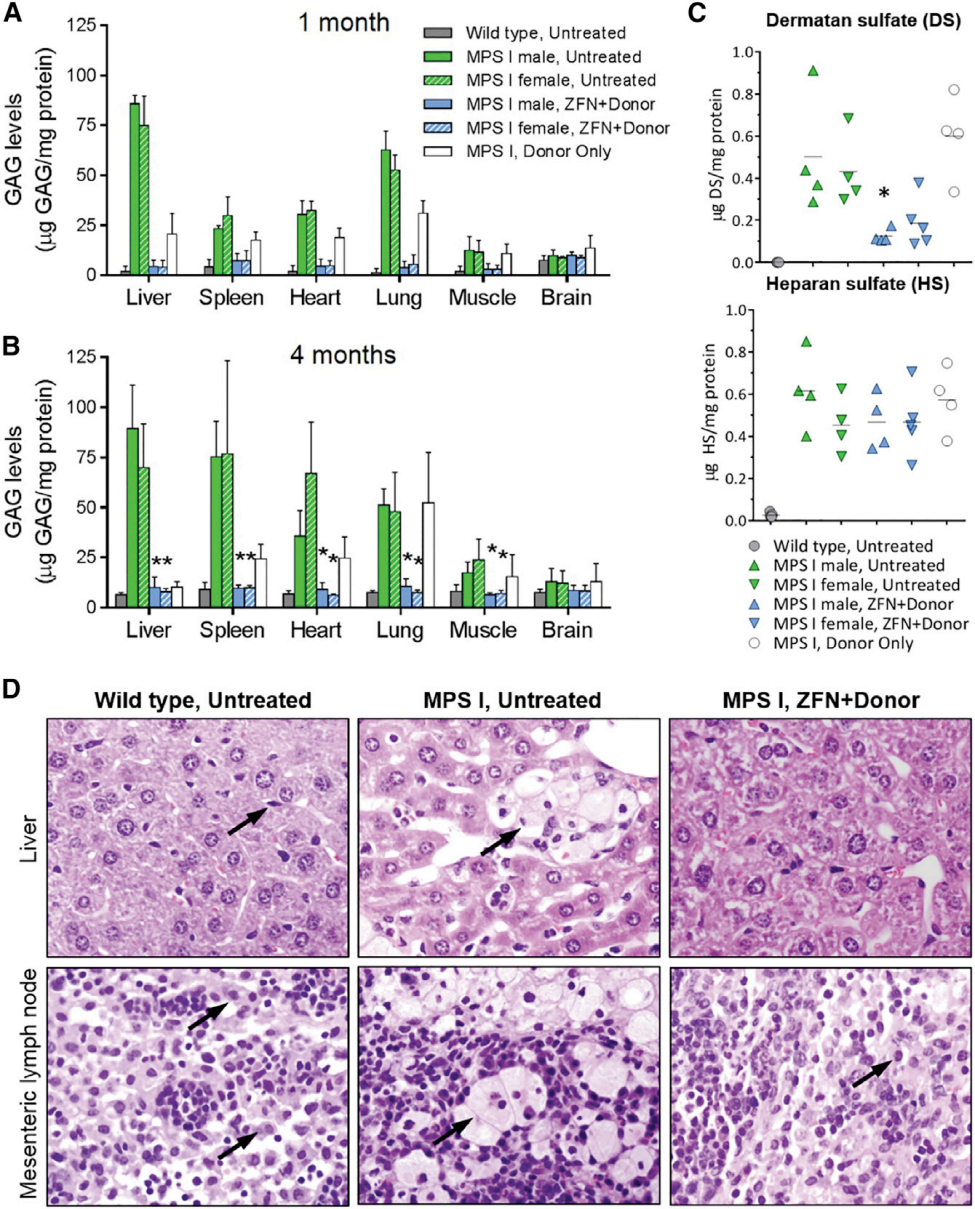
Decreased GAG Storage in ZFN+Donor-Treated MPS I Mice GAG levels present in the indicated tissues at 1 (A) or 4 (B) months post-treatment. Data are shown as mean ± SD. *p < 0.05, when compared to the gender-matched untreated MPS I group. n = 3 per group at 1 month and n = 4–5 per group at 4 months. (C) Levels of dermatan and heparan sulfate in mouse brain homogenates, 4 months post-treatment. Lines indicate mean of each group. All wild-type mice had levels of dermatan sulfate below the lower limit of quantification for this assay (<0.005 μg DS/mg protein lysate). *p < 0.05 for treated versus untreated male MPS I mice. For female MPS I mice, p = 0.0635 when comparing treated and untreated groups. (D) Images taken at 40× magnification from H&E-stained slides indicating the presence or absence of disease-related, highly vacuolated tissue-resident macrophages. Location of tissue resident macrophages indicated by black arrows.

Upon determination that hIDUA was being secreted from the livers of treated mice in a form competent for uptake by distal, secondary tissues, a critical factor in examining the potential of this strategy was to assess whether the levels of hIDUA uptake by secondary tissues were sufficient for reduction of the storage material GAG, which are the natural substrate for IDUA *in vivo* and whose accumulation is a hallmark of MPS I. In this study, the levels of IDUA activity observed in issues of ZFN+donor-treated MPS I mice were sufficient for the complete correction of GAG accumulation in peripheral tissues. Reduction of GAG was evident by 1 month following ZFN+donor treatment, both in the liver (where IDUA is produced from the albumin locus) and in secondary tissues such as the spleen, heart, lung, and muscle, which are dependent on uptake of IDUA from circulation (Figure 4A). Significant reduction of GAG levels was further maintained through 4 months in all examined peripheral tissues (Figure 4B). Slight reduction of GAG levels was also observed in some tissues of mice treated with the hIDUA donor alone. However, reductions were not observed in all tissues from animals administered hIDUA donor alone and were not associated with decreased tissue vacuolation or increased tissue IDUA activity. Urinary excretion of GAGs was also assessed in all mice over the course of 4 months following dosing. Although this assessment yielded significant variability, urinary GAG levels in both male and female ZFN+donor-treated MPS I mice were normalized by day 14 and remained low for the 4-month duration of the study (Figure S3).

At both 1 and 4 months, untreated MPS I control mice displayed only minimal accumulation of GAG in the brain, making it difficult to determine whether IDUA expression in treated mice had any effect in this tissue. As the brain is enriched in chondroitin sulfate^19^—a sulfated GAG that is not a target of IDUA degradation and whose levels are not MPS I related—and the dye-binding assay used here measures total levels of sulfated GAG, a more specific assay may be needed to better distinguish between wild-type and MPS I mice in this tissue. When brain-tissue homogenates were measured specifically for dermatan and heparan sulfate levels by mass spectrometry, there was a significant reduction of dermatan sulfate levels in male ZFN+donor-treated mice as compared to their gender-matched controls at 4 months post-injection (Figure 4C). Female treated mice also showed a trend toward decreased dermatan sulfate levels (p = 0.0635). However, no decreases were observed for heparan sulfate.

The accumulation of GAG in the lysosomes of human MPS I patients and MPS I mice leads to characteristic microscopic lysosomal vacuolation.^12^, ^20^ As part of the histopathological evaluation, the extent of tissue vacuolation was assessed in a subset of tissues from the 1-month interim necropsy (brain, heart, lung, liver, skeletal muscle, and spleen) and in a full list of tissues for the terminal necropsy at 4 months. At necropsy, slides from formalin-fixed, paraffin-embedded tissues from all mice were stained with H&E and examined by a board-certified veterinary pathologist (Seventh Wave Laboratories).

The mice used in this study were 4–10 weeks old at dosing, and thus were ∼8–14 weeks old 1 month post-dosing and ∼21–27 weeks old at 4 months post-dosing. The characteristic natural history of disease in these mice was evident in the microscopic evaluation of tissues, with the pathological findings of increased vacuolation in the untreated MPS I mice being less severe at the 1-month necropsy compared to the 4-month necropsy. Although tissue collection was limited for the interim necropsy, some MPS I mouse tissues had vacuolation of interstitial cells. ZFN+donor-treated mice had a variably decreased incidence of vacuolated interstitial cells in the heart or skeletal muscle, Kupffer cells in the liver, or splenic macrophages or histiocytes. Although decreased in some instances 1 month post-treatment, the presence of vacuolation was considered to be an incomplete response to ZFN+donor treatment.

As expected, tissues from untreated MPS I mice at 4 months displayed the characteristic increased cellular vacuolation, visible as distended cells with greatly enlarged, “foamy” cytoplasm (Figure 4D, arrowheads; compare untreated MPS I and wild-type mouse samples). By contrast, the incidence and severity of these heavily vacuolated cells was greatly reduced in ZFN+donor-treated mice (Figure 4D, “MPS I, ZFN+Donor”). When compared with MPS I control mice, ZFN+donor-treated mice displayed decreased incidence and/or severity of vacuolation in liver, mesenteric lymph node, lungs, heart valve, aortic tunica media, corneal stromal and endothelial cells, and neuronal and glial cells of the spinal cord, along with a large number of other tissues (see Table S1 for a full list of tissues). Histopathological examination further demonstrated that ZFN+donor treatment of MPS I mice was well tolerated with no evidence of clinical signs of toxicity and that ZFN+donor-mediated clearance of lysosomal storage material did not result in adverse pathological findings. These combined data demonstrate that IDUA produced from the albumin locus is being taken up by secondary tissues at levels sufficient for reduction the GAG storage material.

### Prevention of Neurobehavioral Deficit in ZFN+Donor-Treated Mice

**Figure 5:**
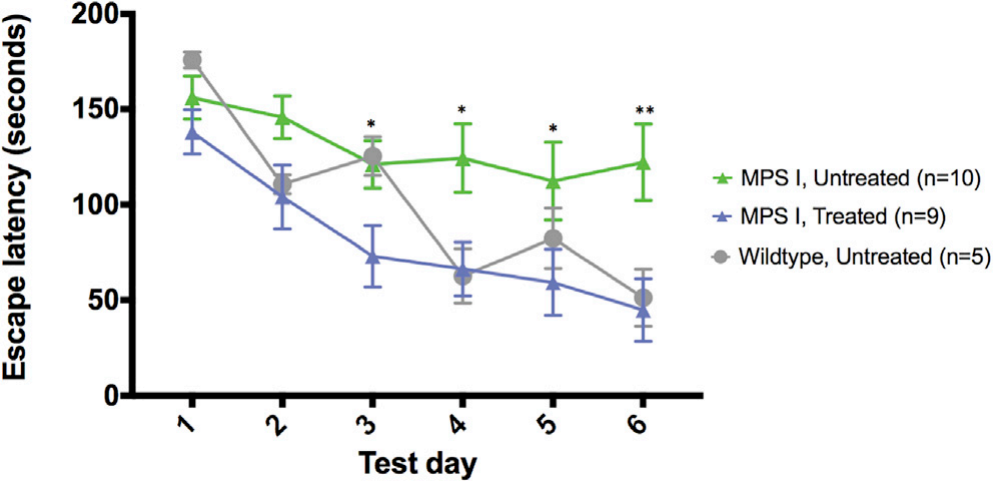
Prevention of Neurobehavioral Deficit in ZFN+Donor-Treated MPS I Mice Performance in Barnes maze is given as time to escape maze in seconds. Data are shown as mean ± SEM at each time point, for MPS I untreated compared to ZFN+Donor treated, significance is *p < 0.05, **p < 0.01.

To determine whether ZFN-mediated hIDUA insertion at the albumin locus and the subsequent supraphysiological levels of IDUA plasma enzyme activity conferred any cognitive benefit in MPS I mice, animals were subjected to Barnes maze testing in the week prior to the 4 month necropsy. The Barnes maze is a test of the ability of the mice to learn and memorize the location of an escape hole on a raised platform. Over the course of 6 days of testing, the average escape latency of control wild-type mice decreases from 176 to 51 s, indicating normal cognitive function. By contrast, untreated MPS I mice show reduced cognitive ability, as evidenced by slower escape times in this test (Figure 5, green symbols). Surprisingly, ZFN+donor-treated MPS I mice perform indistinguishably from wild-type mice and escape from the Barnes maze significantly faster than untreated MPS I mice on days 3–6 of testing. These unexpected results indicate that hIDUA secreted from the liver, in the absence of any BBB-penetrant moiety, may confer significant cognitive benefits to MPS I mice and that small levels of enzyme in the brain may be sufficient for prevention of onset of cognitive deficits in this mouse model.

## Discussion

The data shown here demonstrate that *in vivo* ZFN-mediated insertion of a therapeutic hIDUA transgene at the albumin locus in hepatocytes drives the expression of hIDUA in the liver, secretion of hIDUA into the bloodstream in an active form, and uptake by secondary tissues at levels sufficient for the reduction of GAG storage material. IDUA expression and GAG reduction was sustained over the 4-month course of this study, indicating the durability of this approach following a single intravenous (i.v.) dose administration. Further supporting this durability, an analogous study in which the hF9 gene was inserted at the albumin locus demonstrated that human factor IX (hF9) expression was stable through 60 weeks following treatment.^7^ The insertion and long-term expression of hIDUA from albumin in MPS I mice was well tolerated, with no evidence of treatment-associated clinical observations or adverse macroscopic or microscopic histopathological findings. In addition, the ZFN+donor-mediated clearance of lysosomal storage material did not result in adverse pathological findings in MPS I mice. Additionally, we observed no incidences of hepatocellular carcinoma (HCC), which has been previously associated with AAV-based gene therapy in mice.^21^ In the referenced study, neonatal mice were treated with AAV vectors, and HCC was associated with insertion at a locus not conserved in humans. Additionally, incidence of HCC was influenced by promoter choice in the AAV vector, and no HCC was observed when the hAAT promoter was used to drive gene expression. In the present study, ZFN expression was driven by the hAAT promoter, and the hIDUA donor construct was promoterless, potentially explaining the lack of HCC observed here as compared to other studies. Additionally, the insertional mutagenesis and subsequent oncogenesis observed previously in the clinic using alternate, integrating viral vectors^22^ is safeguarded against in the current approach through (1) the use of a non-integrating virus for transgene delivery, (2) the lack of a promoter in the donor construct, and (3) the precise targeting of a pre-determined “safe harbor” locus (albumin) through the use of sequence-specific ZFNs.

Current standard of care for MPS I patients is ERT. Although ERT has been successful in treating some of the peripheral symptoms of MPS I, there remains significant clinical unmet need, as clearance of GAG in all tissues and the correction of some disease manifestations, such as skeletal abnormalities, cardiac valvular disease, and CNS-related symptoms, is absent or incomplete with ERT.^2^, ^23^ Additionally, ERT imposes significant burden on patient lifestyle, as the patient must receive lifelong weekly, multi-hour infusions of replacement enzyme. By contrast, we propose an *in vivo* ZFN-mediated genome-editing approach in which a functional copy of the hIDUA gene is inserted at the liver albumin locus and IDUA enzyme is expressed in the liver, secreted into the bloodstream, and taken up by secondary tissues for cross-correction of GAG accumulation. This permanent modification should allow sustained expression of replacement IDUA from the liver following a single treatment and builds upon our previous work in establishing the liver albumin locus as a “safe harbor” site for therapeutic transgene insertion and expression.^7^ Sustained, consistent plasma enzyme levels may also facilitate more efficient enzyme uptake by tissues with lower M6P receptor expression due to the constant availability of enzyme for uptake, in contrast to the peak-trough pattern of plasma enzyme levels observed in the current ERT treatment regimen. Additionally, targeted transgene insertion solves the problem of vector washout associated with non-integrating, episomal-based AAV gene therapy,^13^, ^14^, ^15^ as the inserted transgene will be replicated along with the cellular genomic DNA rather than diluted out during cell division. This is of particular importance in the liver-directed treatment of pediatric disease, as there is significant hepatic growth and cellular division (and potential for washout of episomal AAV) during growth and development.^24^ In both normal post-natal liver growth and in liver regeneration after partial hepatectomy, existing hepatocytes are responsible for most, if not all, cellular replication without the activation of a progenitor cell compartment.^25^, ^26^, ^27^ As such, the approach described here, in which ZFN expression is driven by a hepatocyte-specific promoter, is likely to have a long-lasting effect throughout liver maintenance and hepatocyte replication. Finally, the strategy described here relies on the strong, endogenous albumin promoter for therapeutic transgene expression, which should allow for therapeutic levels of enzyme expression from a smaller proportion of modified alleles as compared to targeting the endogenous IDUA locus. This strategy also obviates the need for a promoter to be included in the targeting construct and increasing the effective carrying capacity of the AAV vector.

The behavioral correction of ZFN+donor-treated MPS I mice in this study is a surprising finding. Although heparan sulfate was not reduced, dermatan sulfate was reduced in the treated MPS I mice (and also in treated MPS II mice^28^), thus demonstrating a biochemical response across the BBB. There is a historical generalization that heparan sulfate accumulation (MPS I, II, and III) is associated with mental retardation in patients, while dermatan sulfate accumulation (MPS VI) is not associated with progressive cognitive decline. The mechanisms for seemingly linked association of heparan sulfate and impaired cognition have not been elucidated, although an interesting mechanism was recently proposed.^29^ However, multiple other causes have been suggested. Nevertheless, the reproducible behavioral correction with reduction of dermatan sulfate together indicate that when a constant supply of enzyme is present in the bloodstream at high levels, a small amount may be able to cross the BBB into the central nervous system. The exact mechanism of hIDUA potentially crossing the BBB is unclear. However, the ability of conventional ERT drugs to cross the BBB has been observed previously in the MPS I model used here^17^ as well as in mouse models of MPS II^30^ and MPS VII.^31^ These studies utilized ERT doses 10- to 20-fold higher than standard ERT patient doses on a mg/kg basis. Data from the MPS VII study further indicated that long-term exposure to high levels of enzyme may help to facilitate BBB crossing. Moreover, previous study showed that the difference between Hurler syndrome (cognitive decline) and Scheie syndrome (normal intellect) is only a difference of 0 versus 0.05% of normal enzymatic activity in cultured human fibroblasts.^32^ This implies that a therapy for MPS I may prevent brain disease if it achieves only 0.05% of normal enzyme (in every key cell) across the BBB. Therefore, it is possible that the sustained high level of IDUA observed in the plasma in the current study similarly might similarly allow some IDUA to cross the BBB, resulting in prevention of the onset of behavioral symptoms, although it is unclear at present how this might translate clinically.

The data shown here provide strong support for a ZFN-driven, *in vivo* gene-editing approach for the long-term treatment of peripheral symptoms of MPS I. This strategy is a broadly applicable platform approach, and we have previously demonstrated functional expression of the proteins deficient in hemophilia A and B, MPS II, Fabry disease, and Gaucher disease from the albumin locus following ZFN-mediated insertion.^7^ The current approach can be adapted to alternate model species by using appropriate homology arms on the therapeutic transgene donor construct and designing a pair of ZFNs targeting the corresponding site at intron 1 of albumin. Alternate clinical applications could also be targeted by replacing the corrective transgene encoded on the donor construct with one that is deficient in a given monogenic disease. For MPS I, Sangamo Therapeutics has developed ZFNs targeting intron 1 of the human albumin locus and a hIDUA donor construct with homology arms specific to the human albumin locus delivered using AAV6 vectors. Based on the results described here and additional pre-clinical data, a phase I clinical study has been approved by the FDA (ClinicalTrials.gov: NCT02702115). Further work is being performed to expand this treatment paradigm to additional monogenic diseases.

## Materials and Methods

### Study Design

The MPS I mouse model (*idua*^−/−^) was a kind gift from Dr. Elizabeth Neufeld, UCLA.^12^ All animal care and handling procedures were in compliance with the Institutional Animal Care and Use Committee (IACUC) of the University of Minnesota. Three individual recombinant AAV2/8 vectors (comprised of AAV2 ITRs and the AAV8 capsid) encoding a pair of ZFNs and the hIDUA donor were formulated to a total of 200 μL per mouse in PBS supplemented with 35 mM NaCl and 5% glycerol prior to tail-vein injection. Mice labeled as untreated were dosed with 200 μL of formulation buffer in the absence of vector. Prior to injection, mice were randomized and assigned to groups as indicated in Table 1 to match age and body weight for each gender and were dosed between 4 and 10 weeks of age. Animals were administered cyclophosphamide to suppress a possible immunogenicity response to the hIDUA protein. All mice received a 200 μL intraperitoneal (i.p.) injection of cyclophosphamide (120 mg/kg) on the day before dosing and weekly thereafter through Week 12. After Week 12, the mice received 120 mg/kg once every 2 weeks until necropsy. This change in frequency of administration was due to observations in a few mice of mild alopecia near the injection site and acute mild bradykinesia, which are known side effects of cyclophosphamide. Previous studies have demonstrated efficient immunosuppression to attenuate loss of hIDUA expression in MPS I mice using a bimonthly injection schedule.^33^ Blood samples were collected via the submandibular vein into tubes containing heparin and processed to plasma. Urine samples were collected by gently applying pressure to the urinary bladder.

**Table 1:** Group Descriptions and Dose Levels

Group	Group Description	Mouse Strain	No. of Animals		Each ZFN Dose Level (vg/Mouse)	Donor Dose Level (vg/Mouse)	Total AAV Dose (vg/Mouse)
			Male	Female			
1	formulation buffer control (untreated)	C57BL/6 wild type	8	0	0	0	0
2	formulation buffer control (untreated)	MPS I	8	0	0	0	0
3	formulation buffer control (untreated)	MPS I	0	8	0	0	0
4	ZFNs+donor	MPS I	8	0	1.5e11	1.2e12	1.5e12
5	ZFNs+donor	MPS I	0	8	1.5e11	1.2e12	1.5e12
6	donor only	MPS I	4	4	0	1.2e12	1.2e12

MPS I or wild-type control mice were dosed with a total of 200 μL of formulated AAV vector or control buffer between 4 and 10 weeks of age via a single tail-vein injection.

### IDUA Donor and ZFN Reagents and Vectors

The promoterless hIDUA donor vector targeting the mouse albumin locus (Figure 1A) contains a splice acceptor site derived from exon 2 of human coagulation F9, a codon-optimized hIDUA gene (DNA2.0, Newark, CA) lacking the first 84 nucleotides that encodes the endogenous IDUA signal peptide, and containing a C-terminal myc-FLAG tag and a bovine growth hormone polyadenylation (poly(A)) sequence. This cassette is flanked by a total of approximately 600 bp homology to the ZFN target site at intron 1 of the mouse albumin locus. The ZFNs targeting the mouse albumin locus (48641/31523) have been previously described^7^ and function as obligate heterodimers containing the ELD:KKR mutations in the FokI domain.^34^ AAV vectors used in this study were produced and titered as previously described,^7^ using a HEK293 triple-transfection method in 10-layer CellSTACK Cell Culture Chambers (Corning).

### Quantification of ZFN Modification

Levels of modification (indels) were determined by paired-end deep sequencing on an Illumina MiSeq system (Illumina). Following genomic DNA isolation, the ZFN target locus at intron 1 of albumin was PCR amplified, barcoded, and subjected to MiSeq analysis (forward primer, 5′-ACACGACGCTCTTCCGATCTNNNNAAATCTTGAGTTTGAATGCACAGAT-3′; reverse primer 5′-GACGTGTGCTCTTCCGATCTTTCACTGACCTAAGCTACTCCC-3′). Paired sequences were aligned and merged using SeqPrep software (https://github.com/jstjohn/SeqPrep) and then compared with the wild-type sequence using a Needleman-Wunsch algorithm for sequence alignment^35^ to map indels. ZFN activity is reported as the proportion of sequenced amplicons that differ from wild-type due to indels (% indels).

### IDUA Enzyme Activity

IDUA enzyme activity was determined in a fluorometric assay using the synthetic substrate 4-methylumbelliferyl α-L-iduronide (4-MU-iduronide; Glycosynth) as previously described.^36^ Prior to analysis, tissues were homogenized in PBS using a Polytron Homogenizer (Kinematica). Then, Triton X-100 was added to a final concentration of 0.1%. Tissues were incubated on ice for 10 min, cleared by centrifugation, and protein concentrations were determined using the Pierce Protein Assay Reagent (Thermo Fisher Scientific). Data are shown as nmol 4-MU released per hour, per mL of plasma or mg of protein.

### IDUA Western Blot Analysis

Livers were homogenized in RIPA buffer on a FastPrep-24 instrument (MP Biomedicals) and protein concentration was determined via the Pierce bicinchoninic acid (BCA) Protein Assay Kit (Thermo Fisher Scientific) prior to western blot detection using a LI-COR Odyssey scanner (LI-COR Biotechnology, Lincoln NE). Antibodies used were as follows: IDUA (AF4119, R&D Systems); glyceraldehyde 3-phosphate dehydrogenase (GAPDH) (A00191, GenScript; conjugated to DyLight 800 using the Lightning-Link conjugation kit [Novus Biologicals]).

### Tissue GAG Levels

Levels of GAG in tissues were determined using the Blyscan Glycosaminoglycan Assay Kit (Biocolor Life Science Assays, Accurate Chemical, NY), according to the manufacturer’s instructions. Prior to GAG assay, mouse tissue homogenates were incubated with proteinase K (20 mg/mL) at a ratio of 1:3 at 55°C for 24 hr, followed by heat inactivation of the proteinase K. Tissue homogenates were further digested with 200 units of DNase and 2 μg of RNase at room temperature for 24 hr while shaking. DNase and RNase were heat inactivated and the tissue homogenates were used as input for quantification of GAG levels.

Mass spectrometry of brain tissue homogenates for levels of dermatan and heparan sulfate was performed at Pacific BioLabs (Hercules, CA). In brief, tissues were homogenized in 200 mM ammonium acetate, 20 mM calcium acetate, 4 mM DTT (pH 7.0). Protein concentrations were determined by BCA Protein Assay Kit (Thermo Fisher Scientific), and samples were normalized to 1 mg/mL. Heparan sulfate samples were digested with heparinase I and III, yielding two major disaccharides (Δ-UA-GlcNAc [IV-A] and Δ-UA-GlcNS [IV-S]). Dermatan sulfate samples were digested with chondroitinase B, yielding three major disaccharides (ΔUA-GalNAc (4S) [di-4S], ΔUA-GalNAc (6S) [Δdi-6S], and ΔUA-GalNAc (4S, 6S) [Δdi-4S,6S]). After digestion, an internal standard was spiked in, large molecules were removed by centrifuging through a 10,000 nominal molecular weight limit (NMWL) spin filter or filter plate, and samples were subjected to reversed-phase high-performance liquid chromatography (HPLC). Analytes and internal standards were detected via electrospray ionization tandem mass spectrometry (ESI-MS/MS) using a specific multiple-reaction-monitoring method in negative mode. The peak areas of each analyte and internal standards (ISs) were integrated in their respective MS chromatogram, and the ratios of peak areas were used for quantitation. Concentrations of respective disaccharides in samples were interpolated from a calibration curve of standards of known concentration and are reported in μg/mL, with a lower limit of quantitation (LLOQ) of 0.005 μg/mL for each disaccharide. Disaccharide concentrations were summed for each sample and plotted relative to homogenate input.

### Histology and Histopathology

At the end of study (4 months), animals were necropsied at the University of Minnesota by Seventh Wave Laboratories (Maryland Heights, MO), and tissues or tissue sections were collected and placed into 10% neutral-buffered formalin for fixation. Animals were perfused with 35 mL 1× PBS (pH 7.4) before collection of the organs. The following tissues were trimmed to produce histological sections: adrenal glands, aorta, brain, cecum, cervix, colon, duodenum, epididymis, esophagus, eyes, femoral-tibial joint, gallbladder, Harderian gland, heart, ileum, injection site, jejunum, kidney, larynx, liver, lungs with bronchi, lymph node (mesenteric), optic nerve, ovaries, pancreas, parathyroid gland, pituitary gland, prostate, rectum, salivary gland, sciatic nerve, seminal vesicles, skeletal muscle (quadriceps femoris), skin, spinal cord (cervical, lumbar, thoracic), spleen, sternum with bone marrow, stomach, testes, thymus gland, thyroid gland, tongue, trachea, urinary bladder, uterus, and vagina. Tissues listed above, from animals in all dose groups, were formalin-fixed and then routinely processed and embedded in paraffin blocks. The blocks were sectioned and slides were stained with H&E. Slides were evaluated microscopically by a board-certified veterinary pathologist.

### Barnes Maze

Mice were tested for cognitive performance in the Barnes maze in the final week prior to necropsy at 4 months. The Barnes maze is a circular platform measuring approximately 4 feet in diameter with 40 holes equally spaced around the perimeter. All of the holes are blocked except one to allow the mouse to escape the platform. Visual cues were attached to each of the four walls for the mouse to use in spatial navigation. Mice were placed in the middle of the platform with an opaque funnel covering the mouse. The cover was lifted, releasing the mouse and exposing it to a bright light, triggering the mouse to find the escape hole. Mice were assessed during four trials per day on six consecutive days, with a maximal escape time limited to 3 min. Data were collected and analyzed using the EthoVision program (Noldus).

### Statistical Analyses

All statistical tests were performed using GraphPad Prism (GraphPad Software). For tissue IDUA activity assays and GAG measurements, a Mann-Whitney test was performed on gender-matched treated and untreated groups of MPS I mice. For plasma IDUA activity and Barnes maze, significance was determined by repeated-measures two-way ANOVA, followed by Dunnett’s multiple comparisons test. For all tests, a significance cutoff of p < 0.05 was used.

## Author Contributions

L.O., R.C.D., K.E.M., M.C.H., R.S.M., T.W., and C.B.W. designed the experiments. L.O., R.C.D., M.R., S.T., R.R., S.J.S.M., Y.S., S.S., M.J.P., B.L.K., K.M.P.-P., K.L., and R.D.C. generated reagents, performed the experiments, and analyzed data. L.O., R.C.D., K.E.M., M.C.H., T.W., R.S.M., and C.B.W. wrote and revised the manuscript.

## Conflicts of Interest

The authors declare no competing interests.

## Supplemental Information

Document S1. Figures S1–S3, Table S1, and Supplemental Materials and Methods

Document S2. Article plus Supplemental Information
