# Supplementary material for: ZFN-Mediated In Vivo Genome Editing Corrects Murine Hurler Syndrome
Source: Mol Ther. 2018 Nov 1;27(1):178–87. doi: 10.1016/j.ymthe.2018.10.018 (PMC6319315; doi:10.1016/j.ymthe.2018.10.018)
Supplement: Document S1. Figures S1–S3, Table S1, and Supplemental Materials and Methods [file mmc1.pdf]

YMTHE, Volume *27*

Supplemental Information

ZFN-Mediated *In Vivo* Genome Editing Corrects Murine Hurler Syndrome

Li Ou, Russell C. DeKelver, Michelle Rohde, Susan Tom, Robert Radeke, Susan J. St. Martin, Yolanda Santiago, Scott Sproul, Michelle J. Przybilla, Brenda L. Koniar, Kelly M. Podetz-Pedersen, Kanut Laoharawee, Renee D. Cooksley, Kathleen E. Meyer, Michael C. Holmes, R. Scott McIvor, Thomas Wechsler, and Chester B. Whitley

SUPPLEMENTAL MATERIALS

SUPPLEMENTAL FIGURES AND TABLES

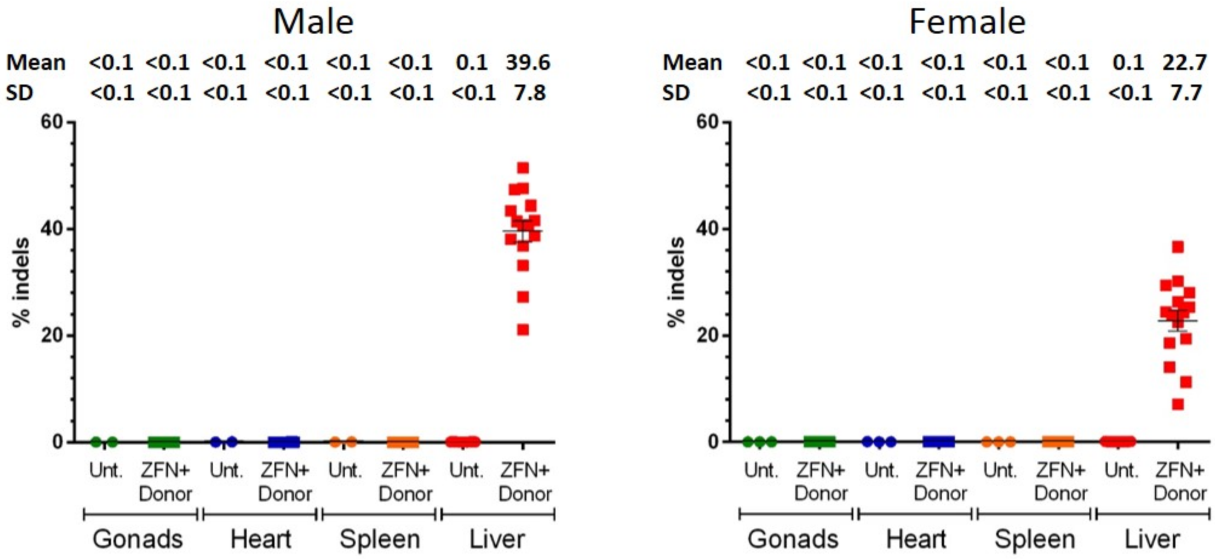

Fig. S1. ZFN activity is restricted to the liver *in vivo*. Male and female wild type C57BL/6 mice were injected with formulation buffer alone as a control (Untreated: 'Unt.') or a combination of three recombinant AAV2/6 vectors encoding albumin-targeting ZFNs 48641 & 31523 under control of the hAAT promoter and the hIDUA donor with homology to intron 1 of the mouse albumin locus (ZFN+Donor), and cohorts were necropsied at 6 months post-injection. AAV doses were 2e11 vg/mouse each ZFN and 1.6e12 vg/mouse donor (male) or 3e11 vg/mouse each ZFN and 2.4e12 vg/mouse donor (female). ZFN activity (% indels) was assessed in genomic DNA extracted from the gonads, heart, spleen and liver. No ZFN activity was observed outside of the liver in either gender.

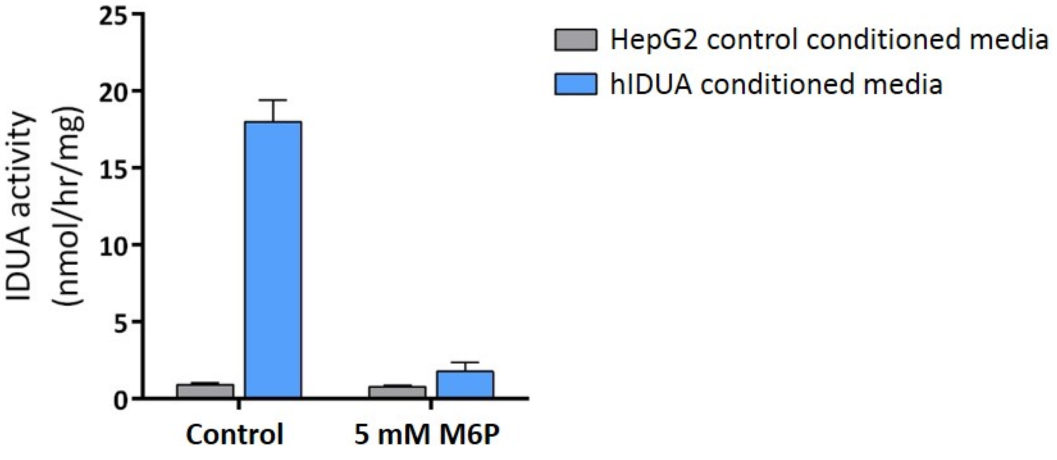

Fig. S2. Excess free mannose-6-phosphate (M6P) blocks uptake of hIDUA produced from the albumin locus. hIDUA enzyme activity in protein extracts from cells incubated with control or hIDUA-conditioned media in the presence or absence of 5 mM M6P.

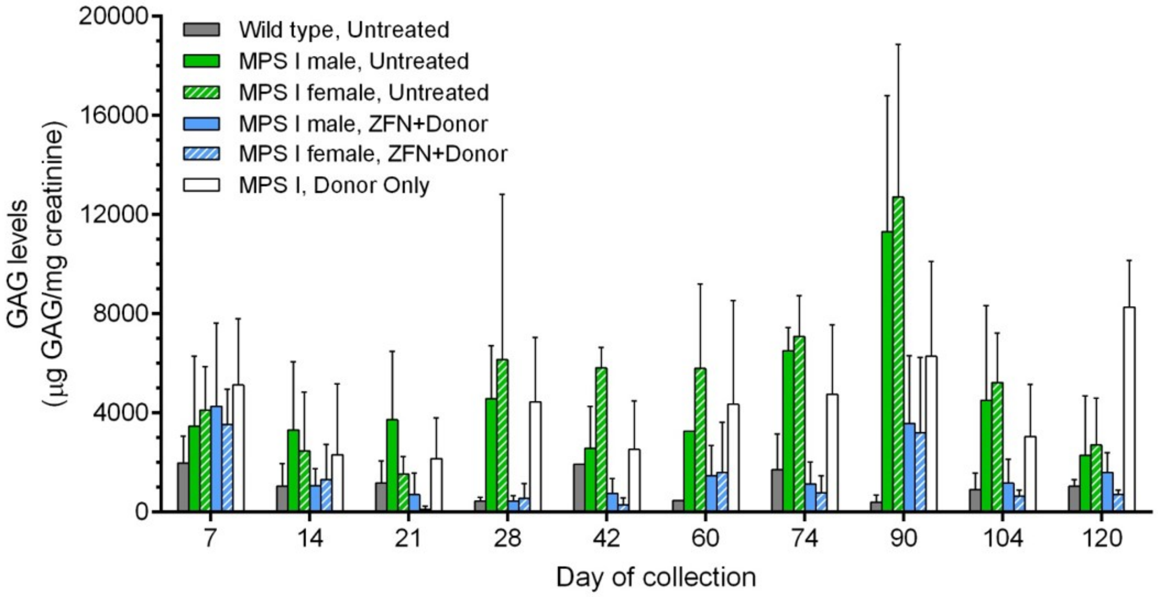

Fig. S3. Decreased urinary glycosaminoglycan (GAG) levels in ZFN+Donor treated MPS I mice. Urinary GAG levels at the indicated time points post-treatment are shown as as mean ± standard deviation.

Male & Female mice	Male mice only	Female mice only
Aortic tunica media, lungs, heart valve and interstitium, renal tubular epithelium, urinary bladder (urothelium and interstitium), skeletal muscle interstitial cells, splenic macrophages/histiocytes, mesenteric lymph node macrophages/histiocytes, thymic interstitial cells, Kupffer cells, sciatic nerve, parathyroid cells, corneal stromal/endothelial cells (Descemet's membrane), gastrointestinal tract (tunica muscularis and interstitium), pituitary pars distalis, injection site, spinal cord (neuronal and glial cells)	Epididymal epithelial or interstitium cells	Sternal or femorotibial joint chondrocytes/ osteocytes/periosteal cells, femorotibial joint synovium, ovaries, uterus, cervix, and vagina

Table S1. Tissues with decreased incidence and/or severity of cellular vacuolation in ZFN+Donor treated MPS I mice.

SUPPLEMENTAL MATERIALS AND METHODS

Mannose-6-phosphate (M6P) inhibition of IDUA uptake

A copy of hIDUA was inserted at intron 1 of albumin in HepG2/C3A cells (ATCC) using a pair of ZFNs targeting a site in the human gene analogous to the mouse target site at albumin (ZFNs 47171/47931). The human hIDUA targeting construct is identical to the mouse construct described, with the exceptions of approximately 400 bp of total homology to human albumin intron 1 in place of the mouse homologous sequence and lacking the myc-flag tag. A pool of modified cells was single-cell cloned, secretion of active IDUA into the cell culture supernatant was confirmed by

IDUA enzyme activity assay, and conditioned media was generated from control HepG2/C3A cells and a clone with IDUA being expressed and secreted from albumin. K562 cells were grown in control- and IDUA-conditioned media for 24 hr in the presence or absence of 5 mM M6P (Sigma-Aldrich). K562 cell pellets were harvested, washed twice in ice-cold PBS, lysed by sonication, and IDUA enzyme activity determined by the 4-MU-iduronide assay.

Urine glycosaminoglycan levels

GAG levels in urine were determined using the Blyscan Sulfated Glycosaminoglycan Assay (Biocolor Inc). Creatinine levels in urine were assessed using the Creatinine Assay Kit (Sigma-Aldrich). Urine GAG levels were expressed as μg GAG/mg creatinine.
